# Communication of Diagnostic Uncertainty in Primary Care and Its Impact on Patient Experience: an Integrative Systematic Review

**DOI:** 10.1007/s11606-022-07768-y

**Published:** 2022-09-20

**Authors:** Maria R. Dahm, William Cattanach, Maureen Williams, Jocelyne M. Basseal, Kelly Gleason, Carmel Crock

**Affiliations:** 1 Institute for Communication in Health Care (ICH), ANU College of Arts and Social Sciences, The Australian National University, Baldessin Precinct Building, 110 Ellery Crescent, Canberra, ACT 2600 Australia; 2 ANU Medical School, ANU College of Health and Medicine, The Australian National University, Canberra, Australia; 3 Patient Advocate, Sydney, Australia; 4 Discipline of Infectious Diseases & Immunology, Faculty of Medicine and Health, The University of Sydney, Sydney, Australia; 5 Johns Hopkins School of Nursing, Baltimore City, MD USA; 6 Royal Victorian Eye and Ear Hospital, Melbourne, Australia

**Keywords:** diagnosis, uncertainty, primary care, interpersonal communication, doctor-patient relationship

## Abstract

**Background:**

Diagnostic uncertainty is a pervasive issue in primary care where patients often present with non-specific symptoms early in the disease process. Knowledge about how clinicians communicate diagnostic uncertainty to patients is crucial to prevent associated diagnostic errors. Yet, in-depth research on the interpersonal communication of diagnostic uncertainty has been limited. We conducted an integrative systematic literature review (PROSPERO CRD42020197624, unfunded) to investigate how primary care doctors communicate diagnostic uncertainty in interactions with patients and how patients experience their care in the face of uncertainty.

**Methods:**

We searched MEDLINE, PsycINFO, and Linguistics and Language Behaviour Abstracts (LLBA) from inception to December 2021 for MeSH and keywords related to ‘communication’, ’diagnosis’, ‘uncertainty’ and ‘primary care’ environments and stakeholders (patients and doctors), and conducted additional handsearching. We included empirical primary care studies published in English on spoken communication of diagnostic uncertainty by doctors to patients. We assessed risk of bias with the QATSDD quality assessment tool and conducted thematic and content analysis to synthesise the results.

**Results:**

Inclusion criteria were met for 19 out of 1281 studies. Doctors used two main communication strategies to manage diagnostic uncertainty: (1) patient-centred communication strategies (e.g. use of empathy), and (2) diagnostic reasoning strategies (e.g. excluding serious diagnoses). Linguistically, diagnostic uncertainty was either disclosed explicitly or implicitly through diverse lexical and syntactical constructions, or not communicated (omission). Patients’ experiences of care in response to the diverse communicative and linguistic strategies were mixed. Patient-centred approaches were generally regarded positively by patients.

**Discussion:**

Despite a small number of included studies, this is the first review to systematically catalogue the diverse communication and linguistic strategies to express diagnostic uncertainty in primary care. Health professionals should be aware of the diverse strategies used to express diagnostic uncertainty in practice and the value of combining patient-centred approaches with diagnostic reasoning strategies.

**Supplementary Information:**

The online version contains supplementary material available at 10.1007/s11606-022-07768-y.

## INTRODUCTION

As a common first point-of-call, patients in primary care often present without fully developed disease processes.^1–3^ Up to 35% of patients exhibit multiple unexplained physical symptoms^2^ or undifferentiated symptoms affecting various body systems.^4^ The passage of time is often a crucial factor when considering diagnostic uncertainty in primary care as most symptoms resolve within 3 months^5^ while a third of symptoms may never be explained.^6^ Thus, diagnostic uncertainty remains a common and not insignificant concern across primary care environments including general practice/family medicine, general internal medicine and general paediatric medicine. Diagnostic uncertainty has been defined from technical, knowledge, perceptive and communicative perspectives.^7–11^ In 2018, Bhise et al.^12^ defined diagnostic uncertainty from the clinician’s perspective as ‘a subjective perception of an inability to provide an accurate explanation of the patient’s health problem’. From a more communication and patient-centred perspective, diagnostic uncertainty can be also conceptualised ‘as any statement made by a provider that either directly or indirectly indicates uncertainty to a patient’.^9^ Diagnostic uncertainty directly impacts clinical practice through delayed diagnosis and health care overutilisation^12^ and as a significant contributor to diagnostic error across most medical specialities.^13, 14^ Diagnostic error has been defined as ‘the failure to (a) establish an accurate and timely explanation of the patient’s health problem(s) or (b) communicate that explanation to the patient’.^15^ Recently, it has been argued the definition should be expanded to include failure to communicate diagnostic uncertainty to patients.^16^ Misdiagnosis-related harms are often caused by the so-called ‘Big Three’ (major vascular events, infections and cancers), for which the role of diagnostic uncertainty in contributing to diagnostic errors is of particular concern.^17, 18^

Diagnostic errors are often due to inadequate collaboration and communication among clinicians, patients and families.^15^ Communicating uncertainty to patients is crucial as it involves them in the diagnostic process and gives them information required for shared decision-making and informed consent, thus impacting patients’ experience of care.^15, 19, 20^ Effectively communicating diagnostic uncertainty to patients can be challenging for doctors given competing priorities and expectations between patients and clinicians.^21, 22^ Wide variability exists in the degree to which clinicians engage in communicating uncertainty to patients.^23^ Although there are suggested protocols for how to communicate uncertainty*,*^24^ few are evidence-based.^22^ Recent systematic reviews have elucidated communication, management and ethical implications of diagnostic uncertainty in primary care.^13, 21^ Yet, what doctors say when expressing uncertainty, including the most commonly used expressions and how patients experience care faced with such expressions of uncertainty, has not been studied in detail^25^ prompting calls for research into the communicative aspects of diagnostic uncertainty incorporating insights from linguistics and communication research.^26^

Given the prevalence of diagnostic uncertainty, its impact on patient care and lack of evidence base regarding communicative strategies, this review sought to answer (i) how doctors in primary care communicate diagnostic uncertainty, i.e. identify communication strategies and linguistic realisations (verbal and non-verbal linguistic discourse features used to express a strategy in actual speech); (ii) how doctors’ communication strategies affect the patient’s experience of care including patients’ reactions and feelings towards the doctor; and (iii) which symptoms and medical conditions are commonly linked to communication of diagnostic uncertainty in primary care, especially the ‘Big Three’ (major vascular events, infections and cancers).^17, 18^

## METHODS

Following the registered review protocol (PROSPERO CRD42020197624),^27^ we systematically searched MEDLINE, PsycINFO, and Linguistics and Language Behaviour Abstracts (LLBA) using individual search strategies (see Appendix 1 for complete search strategies) combining MeSH terms and keywords in title and abstracts associated with ‘Communication’ AND ‘Diagnosis’ AND ‘Uncertainty’ AND ‘Primary care’ environments and stakeholders (patients and doctors). Searches were performed in September 2020 with no limit to publication dates. Additional studies were identified by handsearching references of included full-text articles with handsearches completed in April 2021. No new articles were included after an additional search in December 2021. We did not use a reference librarian to create the search strategies or to conduct the searches which might have limited our results. Non-English language studies (*n*=142) were excluded during the search.

English language studies were included if they described (i) actual or simulated communication of diagnostic uncertainty in primary care settings and (ii) the experiences and attitudes of doctors and patients towards communication of diagnostic uncertainty. We included original research studies in clinic-based primary care settings (general practice/family medicine, general internal medicine, general paediatric medicine) focused on interactions related to the spoken communication of diagnostic uncertainty between medical students, primary care trainees or specialists and patients, family and/or carers (see supplemental material Appendix 2 for detailed inclusion and exclusion criteria). All quantitative, qualitative and mixed methods studies with empirical evidence were considered for inclusion.

### Study Selection

MRD conducted the search of all databases in September 2020 with a subsequent search in December 2021. Following removal of duplicates and guided by predefined inclusion criteria, MRD and WC independently screened title and abstract of all studies using EndNote. Full texts of studies included by either reviewer were again screened independently by MRD and WC. Cases of dispute for final inclusion were resolved through consensus discussion. Selection of full texts and reporting of findings follows PRISMA 2020 guidelines.^28^

### Data Extraction and Synthesis

Data extraction and interpretation focused on identifying strategies used to communicate diagnostic uncertainty and associated linguistic realisations, as well as the impact of such strategies and realisations on the patient experience. MRD and WC extracted data from the selected full texts, first independently, before sharing findings for cross-checking and interpretation. Extracted data included (a) study characteristics (year, country, design, setting, aims, data collection, data analysis, future research, limitations), (b) participants (number, % female, age, clinician experience, symptoms/medical problems), and (c) communication features of diagnostic uncertainty (definition of diagnostic uncertainty, strategies, linguistic realisation, patient responses, impact on patient experience of care). For all studies, including quantitative or mixed methods studies, MRD and WC extracted data from result and discussion section including direct quotes which were taken from clinical interactions, interview excerpts or which were part of intervention tools (e.g. surveys, vignettes).

Based on the nature of the data and the aims of the review, an a priori decision was made to conduct a qualitative rather than quantitative synthesis. MRD and WC synthesised extracted data using an integrative approach,^29^ incorporating thematic and content analysis^30^ to report and integrate findings by themes instead of study design or methods. Through an iterative approach, MRD and WC developed initial categories and formed themes for all extracted data, first independently before discussing findings to identify and refine relationships between certain categories and themes. Preliminary findings were presented to the whole team for discussion, with disputes resolved through consensus deliberations. We summarised quantitative data using basic descriptive statistics.

### Quality Appraisal

We used a purpose-designed assessment tool (QATSDD tool^31^) that allows the appraisal of quality of heterogenous groups of studies with one tool. Our review includes linguistic studies focused on language and discourse features during clinical interaction. Such studies cannot easily be scored with most established quantitatively focused quality assessment tools. The QATSDD tool has been validated by health services researcher in psychology and sociology and was thus deemed suitable for this review situated at the junction of related disciplines linguistics and health communication research.^31^ MRD and WC independently rated studies using the QATSDD tool^31^ suitable for integrative reviews assessing the quality of and synthesising information from studies with quantitative, mixed and qualitative methodologies.^32^ The QATSDD tool rates quality on a scale of 0–3 across 14 items (quantitative and qualitative studies) and 16 items (mixed method studies). Items included theoretical framework, aims, research setting, sample size, recruitment data, data collection, analytic methods, strengths and limitations. Final quality scores are calculated as a percentage of the maximum total score achievable. MRD and WC reviewed their scores and in cases of dispute reached consensus through discussion.

## RESULTS

### Search Results

**Figure 1:**
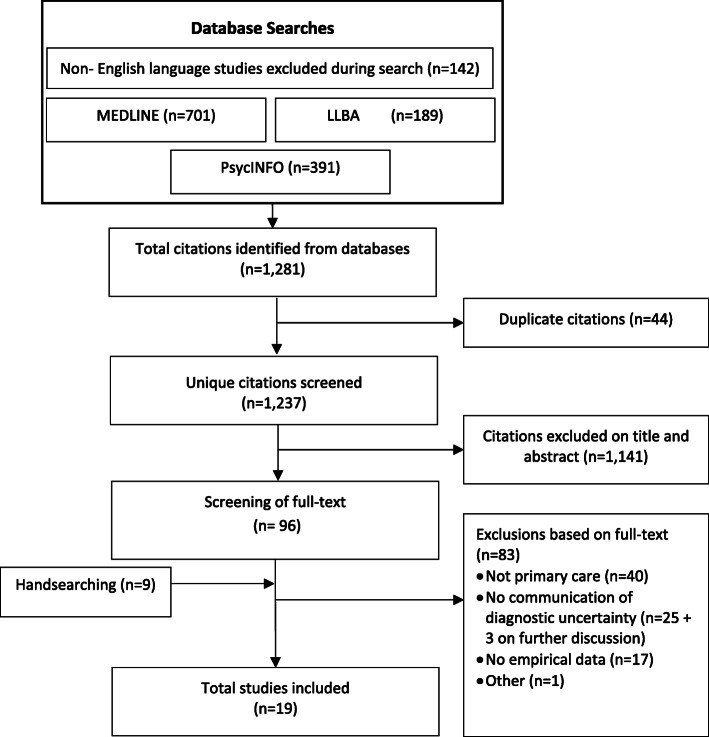
Flow diagram of study identification and study selection.

Our search yielded a total of 1281 studies, with 1237 unique studies screened after removal of duplicates (Fig. 1). Following the review process and handsearching, 19 articles were included in the review (Fig. 1). Three studies^33–35^ initially included after full-text review were excluded during data extraction as they did not yield relevant data related to the communication of diagnostic uncertainty. Included study characteristics and results are summarised in Table 1.

**Table 1:** Summary of Included Studies Characteristics (Author, Year, Setting, Study Design, Participants), Results (Medical Problems Concerned with Uncertainty, Definitions of Diagnostic Uncertainty, Linguistic Realisation, Communication Strategies, and Impact of Communication of Diagnostic Uncertainty to Patients) and Quality Appraisal (QATSSD) Score

Author and year	Location and study setting	Design/method	Number (*n*) of participants (% female (F))	Doctor age (yrs) and experience; Patient age (yrs)	Medical problems concerned with uncertainty (**‘big three’: vascular, infections, and cancers**^18^)	Definitions of diagnostic uncertainty/expressions of uncertainty	Communication strategy and linguistic realisation for diagnostic uncertainty	Impact of communication of diagnostic uncertainty on patient reaction and patient experience of care	QATSSD Score (%)
**Quantitative studies**									
Bhise 2018^36^	USA: convenience sample of parents living in a large US city	Quantitative: experimental vignette-based study design with surveys	Patients (parents) *n*=71 (89% F)	Patient (parent) mean age per intervention group 1. 36.3 yrs 2. 38.8 yrs 3. 41.4 yrs	• Abdominal pain (lasting 3 weeks)	Not given	**Strategy**: • Explicit disclosure • Implicit disclosure • Reasoning (differential diagnosis) **Realisation**: - Negated declarative (e.g. ‘not sure’) - modal verb (e.g. ‘could be caused by’) - Modal adverb/adjective (e.g. ‘most likely’) - Declarative (e.g. ‘could be caused by Disease A vs. Disease B or Disease C’)	**Experience of care:** • Explicit expression associated with less patient trust, adherence, and perceived doctor competence than implicit. • Reasoning favourable strategies (differentials) resulted in better patient experience of care.	73.8
Gerrity 1990^23^	USA: doctors stratified by specialty (e.g. family medicine and internal medicine): half were licensed in North Carolina and half in Oregon.	Quantitative: questionnaire to doctors	Doctor *n* = 428 (12% F)	Doctor mean age: 46 ± 13 yrs Doctor mean experience: 20 ±14 yrs	No mention	Not given	**Strategy**: • Omission • Explicit disclosure **Realisation**: - Negated declarative (e.g. ‘I don’t know’)	Not mentioned	88.1
Gerrity 1992^37^	USA: Doctors stratified by specialty (e.g. family medicine and internal medicine): half were licensed in North Carolina and half in Oregon.	Quantitative: questionnaire to doctors	Doctor *n* = 428 (12% F)	Doctor mean age: 46 ± 13 yrs Doctor mean experience: 20 ±14 yrs	• Abdominal pain • Sore throat • **Chest pain (vascular)**	Not given	**Strategy**: • Omission • Explicit disclosure **Realisation**: Not available	**Patient reaction:** Doctors withholding uncertainty because of potential to dissatisfy or confuse patient.	76.2
Ogden 2002^38^	UK: Six general practices in the south-east of England	Quantitative: cross-sectional design, matched questionnaire	GPs *n* = 66 (42% F) Patients *n* = 550 (69% F)	GPs mean age: 44.86 ± 9.03 yrs Patient mean age: 48.09 ± 17.32 yrs	• No mention	**Expression of uncertainty:** ‘Expression of uncertainty […] were conceptualised as either behavioural expression (e.g. using a book or a computer or referring to hospital) or verbal expressions (e.g. “I don’t know” and “I’m not sure about this”)’ p. 172	**Strategy**: • Explicit disclosure • Implicit disclosure • PCC (reassurance) • Reasoning (information seeking) **Realisations**: - Negated declarative (e.g. ‘I don’t know’, ‘I’m not sure about this’) - Declarative (e.g. ‘I need to find out more’, ‘let’s see what happens’) - Modal verbs (e.g. ‘this *might* be..’) - Introductory phrase (e.g. ‘I think’)	**Experience of care:** • Explicit disclosure was associated with less patient confidence. • Patients rated verbal explicit statements worse than doctors. • Older patients with more experience with doctor tolerated uncertainty better. • Information seeking and implicit forms of expression of uncertainty seen as benign and even positive.	76.2
Olsen 2018^39^	US: The University of Minnesota Paediatric Residency Program (commonly encountered clinical situations in general paediatrics)	Quantitative: Two-phase simulation-based educational intervention	Doctors (residents) *n*=23 Patients (simulated) *n*=4 (50% F)	Doctor experience: 2 yrs (Simulated) Patient age range: 0–6 yrs	• Hypotonia • Features consistent with Trisomy 21 • **Ongoing fever (infection**) • **Viral infection (infection)** • Lymphadenopathy • **Malignancy, leukaemia (cancer)** • Elevated inflammatory markers • Fatigue • Pancytopenia • Abnormal movements and ‘spells’	**Diagnostic uncertainty:** ‘The subjective and often appropriate perception that a clear and accurate explanation of a patient’s health problem is not able to be determined at this point in time.’ p.244 (modified from^12^)	**Strategy**: • Explicit disclosure • PCC (reassurance) • PCC (empathy) • Reasoning (diagnostic process) • Reasoning (eliminate/candidate diagnosis) **Realisation**: Not available	Not mentioned	83.3
Storten-beker 2019^40^	NLD: GP clinic	Quantitative: analysis of video-recorded consultations, pre-post consult questionnaire for patients. Regression modelling	Doctors *n*=18 (N/A) Patients *n* =82 (60% F)	Doctor (GP) age not given Patient mean age: 52.6 (range 18–86)	• Chest pain due to acid reflux (Medically explained symptom) • **Vague chest pain (MUS) (vascular)**	**Expression of uncertainty:** ‘Frequent expressions of implicit uncertainty such as uncertain verbs (e.g. “could”, “I think”), lexical items (e.g. “probably”, “maybe”) and pragmatic particles (e.g. “sort of”)’ p. 2350	**Strategy**: • Implicit disclosure **Realisation**: - Modal verb (e.g. ‘could’) - Modal adverb/adjective (e.g. ‘maybe’, ‘probably’) - Adverb/adjective (e.g. ‘sort of’) - Introductory phrase (e.g. ‘I think’)	**Patient reaction:** • No relationship between implicit uncertainty and patient anxiety.	57.1
**Mixed methods studies**									
Cousin 2013^41^	SWI: Study 1. Vignette: analogue patients, ‘Doctor’ not specified Study 2: Interactions: GPs from the French-speaking part of Switzerland	Mixed: vignette-survey of patients and videotaped actual doctor-patient interactions and patients satisfaction survey	Vignette: patients *n*=120 (50% F) Interaction: doctors (GPs) *n*=36 (44% F) Patients *n* = 69 (47% F)	Vignette: patient mean age: 36.02 ± 12.51 yrs Interaction: doctor (GPs) mean age: 47.18 ± 9.55 yrs Patient mean age: 50.72 ± 18.19 yrs	• Back pain • Regular check-ups • Hypertension	**Expressions of uncertainty:** ‘Direct and indirect verbal expressions of uncertainty. Examples of direct expressions include “I don’t know” and “I have difficulty in answering this question”. Examples of indirect expressions include, for instance, certain adverbs (e.g. “probably”, “maybe”), probability statements (“There’s a good chance that…”), modal verbs (e.g. “might”, “may”, “should”) and conditional sentences (“If you feel better in a week…”)’ p. 927	**Strategy**: • Explicit disclosure • Implicit disclosure **Realisation**: - Negated declarative (e.g. ‘I cannot tell you’) - Modal adverb/adjective (e.g. ‘maybe’, ‘probably’) - Modal verb (e.g. ‘might’, ‘should’) - Conditional (e.g. ‘if you feel better in a week’) - Probability statement (e.g. ‘there’s a good chance that’)	**Experience of care:** Less patient satisfaction associated when female doctor communicated diagnostic uncertainty (no impact with male doctor). This effect only seen with male patients.	77.1
Epstein 2007^42^	USA: family doctors and general internists in Greater Rochester area	Mixed: patient survey and audio recording of doctors interacting with a simulated patient	Doctors *n*=100 (23% F) Patients (survey) *n*=4746 (62% F)	Survey: patient mean age: 45 yrs (range 18–65) Simulated patients: presented as 48-year-old	• **Chest pain** (GERD role or medically unexplained symptoms (MUS) **vascular, ?cancer)** • Fatigue (MUS) • Dizziness (MUS) • Emotional distress (MUS)	Not given	**Strategy**: • Explicit disclosure • PCC (empathy) **Realisation**: - Negated declarative (e.g. ‘I don’t know’)	**Experience of care:** Explicit expression of uncertainty not associated with lower rating of doctor satisfaction/trust/autonomy/support/knowledge.	85.4
Gordon 2000^25^	USA: university-affiliated general medicine clinic. General Medicine Clinic of the Portland, Oregon Veterans Affairs (VA) Medical Centre	Mixed: surveys of clinician response to uncertainty, patient behaviours and satisfaction with use of expression of uncertainty in consultations	Doctor *n*=43 (35% F) Patients *n*=43 (0% F)	Doctor age: not available Patient mean age: 62 yrs (range: 26–78) Doctor experience: 2 yrs (*n*=14), 3 yrs (*n*=14), staff doctors (*n*=15)	• Chronic illness requiring medication	**Expressions of uncertainty:** ‘[…] defined as a direct and unambiguous statement of uncertainty (for example, “I don’t know” or “It’s not clear”).’ p. 61	**Strategy**: • Explicit disclosure • PCC (empathy) • PCC (information giving) Reasoning (eliminate/candidate diagnosis) **Realisation**: - Negated declarative (e.g. ‘it’s not clear’) - Approximator (e.g. ‘pretty much normal’) - Introductory phrase (e.g. ‘my guess is’) - Adverb/adjective (e.g. ‘reportedly’) - Probability statement (e.g. ‘there’s a good chance that’)	**Experience of care:** Doctor explicit expressions of uncertainty were associated with greater patient satisfaction only when using positive talk, partnership building, and information giving. These patients wanted more information, and got more as a result.	58.3
**Qualitative studies**									
Arborelius 1991^43^	SWE: four health care centres	Qualitative: comments on videotaped consultations	Doctors *n*=9 (44% F) Patients *n*=14 (36% F)	Doctor mean age: 40 (range 35–50) Patient age: 20–97 yrs Doctor mean experience: 13 yrs (range 9–27)	• Weight loss • Loss of appetite • Pectoral and shoulder pains • Palpitation of the heart • Muscular rupture • Rheumatism • Hard life situation • Cold • **UTI (infection)** • Rectal pain	Not given	**Strategy**: • Omission **Realisation**: Not available	Not mentioned	33.3
Clarke 2014^44^	UK: tertiary referral centre and GP clinics in Southern England	Qualitative: thematic analysis of qualitative semi-structured interviews	Doctors *n*=9 (N/A) Patients (parents) *n*=21 (86% F)	Not available	• New diagnosis of **acute leukaemia** (**cancer**)	Not given	**Strategy**: • Reassurance (safety netting) • Patient-centred communication (PCC, empathy) • Reasoning (eliminate/candidate diagnosis) **Realisation**: Not available	Not mentioned	83.3
Heath 1992^45^	UK: general practice consultants gathered in various settings throughout the British Isles.	Qualitative: (No further methods stated)	Not given	Not given	• Ulcer • Anxiety • (Wear and tear) arthritis • **Conjunctivitis (infection)**	Not given	**Strategy**: • Explicit disclosure • Implicit disclosure • Embodied action • Reasoning (eliminate/candidate diagnosis) **Realisation**: - Negated declarative (e.g. ‘I wouldn’t know’) - Introductory phrase (e.g. ‘I think’) - Adverb/adjective (e.g. ‘not a *totally typical*’) - Interrogative (e.g. ‘if I was to say to you …?’) - Conditional (e.g. ‘I’m wondering if you’ve got…’) - Intentional vagueness (e.g. ‘you know’) - Hesitation	**Patient reaction:** • Patient does not respond, when doctor provides candidate diagnosis. • Patient responds with own opinion and lay perspective without challenging doctor when doctor presents diagnosis as tentative question. • Patient supports doctor’s diagnosis citing other sources (e.g. spouse).	19.1
Heritage 2019^46^	USA: Western and Southern US clinical practices (2003–2005)	Qualitative: conversation analysis of video-recorded interactions + coding (grounded theory)	Doctor *n*=71 (N/A) Patients *n*= 212 (N/A)	Not given	• Upper respiratory symptoms • Musculoskeletal conditions	**Expression of uncertainty:** ‘[D]iagnoses delivered using epistemic modality, evidentialization, and epidemiologic generalization. […], mitigated diagnoses are just that: named medical conditions presented with some element of epistemic distancing.’ p. 267	**Strategy**: • Explicit disclosure • Implicit disclosure • Reasoning (eliminate/candidate diagnosis) **Realisation**: - Negated declarative (e.g. ‘I hope you don’t have…’) - Generalising declarative (e.g. ‘the most common reason for the lining to be irritated is…’) - Modal verb (e.g. ‘could’, ‘might’) - Perception verb (e.g. ‘looks like’, ‘it feels like’) - Modal adverb/adjective (e.g. ‘likely’, ‘maybe’, ‘probably’) - Impersonal pronouns (e.g. ‘it feels like’) - Introductory phrase (e.g. ‘*what I think* you have is..’) - Intentional vagueness (e.g. ‘you know’) - Gaze	**Patient reaction:** • Patient verbal responses are more extensive when mitigation present in diagnostic statements. • No direct gaze reduces patient likelihood to respond verbally.	42.9
Maynard 2003^47^	USA: internal medicine clinic in hospital	Qualitative: case study ‘single case analysis’, conversation analytic research	Doctor *n*=1 (0% F) Patient *n*=1 (100% F)	Not given	• Mammograph result of **lump (cancer)**	Not given	**Strategy**: • Explicit disclosure • Implicit disclosure • Embodied action • PCC (interpersonal) • PCC (information giving) • Reasoning (diagnostic process) **Realisation**: - Negated declarative (e.g. ‘that’s not a hundred percent’, ‘[but we] can’t even tell: if yer having [X] or not’) - Modal verb (e.g. ‘this could be…’) - Perception verb (e.g. ‘it appears to be…’) - Impersonal pronouns (e.g. ‘it’s kind of like’) - Introductory phrase (e.g. ‘according to the ...’, ‘they see something that …’) - Intentional vagueness (e.g. ‘it’s *kind of* like…’) - Hesitation	**Patient reaction:** • Patient attempts to align understanding in response to intentional vagueness. • In response to hedging, patient is misaligned with doctor focus. • Humour with explicit disclosure allowed patient to accept uncertainty.	26.2
Maynard 2006^48^	USA: Midwest university hospital primary care centre and Eastern US state primary care clinic	Qualitative: conversation-analytic investigations Video, case studies (1 good news, 1 bad news, 2 snippets)	Doctors *n*=3 (N/A) Patients *n*=3 (75% F)	Patient age range: 37–50 (patients 1 and 2) Patients 3 and 4 age not given	• **Severe chest pain (vascular**) • Leg pain • **Armpit lump (cancer)** • (Patient 2 has a definite cancer diagnosis)	**Diagnostic uncertainty:** ‘persistent medical complaints may go unexplained when a serious diagnostic possibility is excluded. This raises the specter of indeterminacy and uncertainty in clinical medicine. […clinicians] can be faced with symptoms of indeterminate origins and consequently must deal with uncertainty about a larger medical picture of the patient surrounding one particular episode of diagnostic news […]’ pp. 250, 276	**Strategy**: • PCC (reassurance) • Reasoning (eliminate/candidate diagnosis) **Realisation**: Not available	**Patient reaction:** • In response to serious diagnosis elimination patient tried to justify their visits because of ongoing symptoms.	23.8
Meyer 2019^52^	USA: paediatric clinicians at two large academic medical institutions in Texas	Qualitative: semi-structured, face-to-face interviews	Doctors *n*=18 (65% F)	Not specified Doctor experience: 0–16 yrs	• Cough • Fever **(?infection**) • Headache • Vomiting • Abdominal pain	**Diagnostic uncertainty:** ‘Subjective perception of an inability to provide an accurate explanation of the patient’s health problem’ p. G108 (adopted from^12^)	**Strategy**: • Omission • Explicit disclosure • Implicit disclosure • PCC (reassurance) • PCC (empathy) • PCC (managing expectations) • PCC (information giving) • Reasoning (diagnostic process) • Reasoning (eliminate/candidate diagnosis) • Reasoning (information seeking) **Realisations:** - Negated declarative (e.g. ‘we don’t know what’s going on’)	**Patient reaction:** • Patients with lower education levels were less engaged with less request for details. Patients with higher education levels were more engagement but had more discomfort with uncertainty. • Patients from some cultural backgrounds [unspecified] regarded uncertain doctors less positively. • Fear, frustration, grief, anxiety in response to uncertainty. Empathy (listening) and planning was used to deal with these emotions. **Experience of care:** • Explicit honest expression of uncertainty led to more trust in doctor.	78.6
Paton 2017^49^	UK: GP clinic	Qualitative: case study	Patient *n* = 1 (0% F)	3-year-old boy	• Wheeze and respiratory symptoms • **Chest infection (infection)**	Not given	**Strategy:** • PCC (reassurance) • PCC (empathy) • PCC (information giving) • Reasoning (diagnostic process) **Realisation**: Not available	**Patient reaction:** • Empathy, explaining the diagnostic process and tailored information giving were strategies used in response to patient parents’ frustration at uncertainty. Parents felt reassured.	23.8
Peräkylä 1998^50^	FIN: four Finnish primary care health centres	Qualitative: conversation analysis of video-recorded interactions	Doctors *n*=14 (N/A) Patients *n* > 100 (N/A)	Not given	• **Joint infection** • **Bacterial infection** • Cartilage injury	Not given	**Strategy:** • Explicit disclosure • Implicit disclosure • Embodied action • PCC (reassurance) • PCC (information giving) • Reasoning (diagnostic process) **Realisation**: - Negated declarative (e.g. ‘but no bacterial infection seems to be there’) - Perception verb (e.g. ‘here appears to be…’, ‘seems to be …’) - Intentional vagueness (e.g. ‘things like that’) - Impersonal pronouns (e.g. ‘it really behaves so much as if..’) - Hesitation	Not mentioned	42.9
Peräkylä 2006^51^	FIN: four Finnish primary care health centres	Qualitative: conversation analysis of video-recorded interactions	Doctors *n*=14 (N/A) Patients *n* > 100 (N/A)	Not given	• **Joint infection** • **Bacterial infection**	Not given	**Strategy**: • Implicit disclosure • PCC (information giving) • Reasoning (diagnostic process) • Reasoning (eliminate/candidate diagnosis) **Realisation**: - Declarative (e.g. ‘It’s probably a bit the…’) - Modal adverb/adjective (e.g. ‘probably’) - Perception verb (e.g. ‘the [X] feels …’, ‘seems to be …’) - Introductory phrase (e.g. ‘As tapping on the vertebrae didn’t cause any pain […] it suggests a …’) - Intentional vagueness (e.g. ‘a bit of …’) - Impersonal pronouns (e.g. ‘it suggests a …’)	**Patient reaction:** • Communication of uncertainty led to longer patient verbal responses (weak association). • Plain assertions led to passive reaction of patient in one case. • Explaining evidence as a way to manage diagnostic uncertainty when discrepancy exist between patient and doctor.	38.1

Abbreviations: *SWE*, Sweden; *GERD*, gastroesophageal reflux disease; *GP*, general practitioner; *MUS*, medically unexplained symptom(s); *US*, United States; *UK*, United Kingdom; *PCC*, patient-centred care; *SWI*, Switzerland; *FIN*, Finland, *NLD*, Netherlands

Study designs included quantitative,^23, 36–40^ mixed methods,^25, 41, 42^ and qualitative.^43–52^ Studies were published between 1991 and 2019 and conducted predominantly in the USA (*n*=10) and UK (*n*=4).

A total of 6876 participants were included in the study: 839 doctors and 6037 patients. Doctors’ years of experience ranged from 1^st^ year post medical school graduation to fully qualified physicians with 34 years of experience (Table 1). Where available, doctor’s mean ages ranged from 40 to 47 years. Excluding case studies, the percentage of female doctors varied from 12%^23^ to 65%.^52^ Patients’ ages ranged from the first year of life^39^ to 86 years.^40^ Excluding case studies, the percentage of female patients varied from 0%^25^ to 89%.^36^ Only a quarter of studies (*n*=5) included some information about patient diversity such as race/ethnicity,^36, 38, 42^ education,^25, 36, 41, 42^ or social class.^38^ The predominant race (between 68% and 87%) of participants was white^36, 38, 42^ and most had completed high school education or higher.^25, 36, 41, 42^

Of the 19 articles, less than half (*n*=8) included definitions for expressions of uncertainty^25, 38, 40, 41, 46^ or diagnostic uncertainty^39, 48, 52^ (frequently adopting or adapting Bhise et al.’s definition^53^). Expressions of uncertainty were defined generally (e.g. ‘verbal expressions of uncertainty’^41^) or specifically^25, 40, 46^ (e.g. ‘direct and unambiguous statement of uncertainty (e.g. “I don’t know” or “It’s not clear”.)’)^25^

### Quality Ratings

Two reviewers reached good^54^ to excellent^55^ agreement (intraclass correlation coefficient = 0.78) rating all studies. Here, we include quality ratings of the more experienced reviewer (MRD). Quality ratings (QATSSD scores) ranged from 19.1%^45^ to 88.1%,^23^ with qualitative studies scoring lowest on average (48%), followed by mixed methods (73.6%) and quantitative studies (75.8%, see supplemental material Appendix 3 for detailed ratings). No studies were excluded based on quality ratings. Across all studies, the lowest average scores were recorded for providing ‘evidence of sample size considered in terms of analysis’ (0.8/3), ‘evidence of user involvement in design’ (0.8/3) and ‘detailed recruitment data’ (1.4/3).

### Common Signs and Symptoms Mentioned in Studies

Infection was the most common disease state identified in the studies associated with diagnostic uncertainty with references to infection, or symptoms of infection (e.g. fever), in 37% (*n*=7) of studies.^39, 43, 45, 49–52^ Other common symptoms included chest pain (*n*=4),^37, 40, 42, 48^ abdominal pain (*n*=3),^36, 37, 52^ neurological (*n*=3, e.g. dizziness or headache)^39, 42, 52^ and respiratory symptoms (*n*=3, e.g. cough or wheeze).^46, 49, 52^

Almost two-thirds (63%, *n*=12) of studies related to the ‘Big Three’^18^ pathologies associated with diagnostic errors (see Table 1). Seven studies mentioned one or more of these pathologies directly,^39, 43–45, 49–51^ and five studies referred to presenting complaints associated with the ‘Big Three’, e.g. chest pain for major vascular events^37, 40, 42, 48^ and ‘lumps’ for cancer.^47, 48^

### Thematic Analysis

#### Communication Strategies and Linguistic Realisations for Diagnostic Uncertainty

We identified two overarching categories of communication strategies used to manage diagnostic uncertainty: (1) patient-centred strategies and (2) diagnostic reasoning strategies. Patient-centred strategies included (i) reassurance (e.g. safety netting, referrals, re-eliciting patient narratives),^38, 39, 44, 48–50, 52^ (ii) empathy (e.g. listening, exploring emotions),^25, 39, 42, 44, 49, 52^ (iii) information giving (e.g. tailored, providing evidence),^25, 47, 49–52^ (iv) managing expectations^52^ and (v) interpersonal skills (humour).^47^ Diagnostic reasoning strategies included (i) commenting on the diagnostic process,^39, 47, 49–52^ (ii) differential diagnosis (e.g. eliminating serious diagnosis or providing candidate diagnosis),^25, 36, 39, 44–46, 48, 51, 52^ and (iii) information seeking (consulting other clinicians, books, internet).^38, 52^

We identified three overarching linguistic strategies to communicate diagnostic uncertainty: (1) explicit disclosure (*n*=13),^23, 25, 36–39, 41, 42, 45–47, 50, 52^ (2) implicit disclosure (*n*=10)^36, 38, 40, 41, 45–47, 50–52^ and (3) omission (*n*=4).^23, 37, 43, 52^ The three overarching strategies had diverse linguistic realisations (e.g. syntactical or lexical structures). Explicit disclosures were exclusively realised through one syntactical structure: negated declaratives^23, 25, 36, 38, 41, 42, 45–47, 50, 52^ (e.g. ‘I don’t know’,^23, 25, 38, 42, 52^ ‘But that’s not a hundred percent as you know.’^47^). Implicit disclosure used diverse linguistic realisations, including different syntactical structures: declaratives^25, 38, 41^ (e.g. ‘I think this might be…’,^38^ ‘There’s a good chance that…’^41^), questions (e.g. ‘If I was to say to you…’^45^), and conditionals^41, 45^ (e.g. ‘If you feel better in a week.’^41^). Implicit syntactical structures were often combined with various lexical structures: modal verbs^36, 38, 40, 41, 46, 47^ (e.g. could, may, should), modal adverbs/adjectives^36, 40, 41, 46, 51^ (e.g. probably, most likely, maybe), perception verbs^46, 47, 50, 51^ (e.g. it feels/looks like’,^46^ ‘it appears to be…’^47, 50, 51^), introductory phrases^25, 38, 40, 45–47, 51^ (e.g. ‘I think’,^38, 40, 45–47^ ‘They see something’,^47^ ‘My guess is…’^25^) and embodied actions^45, 47, 50^ (e.g. hesitations). Five studies did not specify any linguistic realisations.^39, 43, 44, 48, 49^

Omission was used as a conscious strategy by doctors, for example, when ‘clinicians acknowledged they did not always share everything they were uncertain about (e.g. an extensive list of differentials)’.^52^ The reasons why doctors consciously did not disclose diagnostic uncertainty to their patients included doctors lacking diagnostic understanding or clarity,^43^ general reluctance to disclose uncertainty,^23, 37^ doctors believing patients want a clear answer,^37^ and ruling out serious diagnosis without further explanation.^52^

#### Impact of Communicating Diagnostic Uncertainty

Patients’ reactions to what was said and their experience of care were influenced by expressions of diagnostic uncertainty. We included as reactions patient (emotional) responses to diagnostic uncertainty such as engagement, frustration, and anxiety. We considered experience of patient care as what the patient felt about the doctor (e.g. trust and confidence in the doctor). Patients’ reactions and experiences of care were multi-varied and communication strategies had neither entirely positive nor negative impacts. Table 2 provides an overview on reactions and experiences of care concerning the identified communication strategies and linguistic realisations across the included different study designs. In this table, we further separated qualitative studies into those drawing on authentic recorded interaction and those drawing on interviews, because a combination of findings from ‘what people say they do’ in interviews and ‘what they actually do’ in interactions is often needed to gain a more complete understanding of a phenomenon.^56^ Qualitative studies analysing authentic interactions, while generally low on the QATSDD quality rating (see supplemental material Appendix 3), were the only studies that provided findings across all communication strategies and linguistic realisations.

**Table 2:** Summary of Patient Reactions and Experience of Care in Relations to Communication Strategies and Linguistic Realisations Across Study Designs

Study design	Communication strategies		Linguistic realisations		
	Patient centred	Diagnostic reasoning	Explicit	Implicit	Omission
Quantitative		• ↑care experience^36^ • ↑ doctor competence/patient confidence in doctor (seek information from doctors)^38^ • ↓ doctor competence/patient confidence in doctor (seek information from nurses)^38^	• ↓ trust^36^ • ↓ adherence^36^ • ↓ doctor competence/patient confidence in doctor^36^	• ↑ trust^36^ • ↑ adherence^36^ • ↑ doctor competence^36^ • ↑ care experience^38^ • ↓ doctor competence/patient confidence in doctor^38^	• ↓ patient satisfaction^37^ • patient confusion^37^
Mixed methods			• ↑ care experience^25^ • ↓ patient satisfaction (only female doctors)^41^ • ↑ patient satisfaction (only w/ patient centred strategies)^25^		
Qualitative; authentic recorded interaction	• ↑ patient participation^46^ • ↑ acceptance^47^ • ↑ rapport^47^	• ↓ patient participation^45^ • ↑ patient participation^45, 51^ • threatens sick role^48^ • ↑ doctor competence/patient confidence in doctor^51^ ↑ adherence^36, 51^	• ↑ acceptance^47^ • ↑ patient participation^51^	• ↑ patient participation^45, 51^ • ↑ relationship building^45^	• frustration^46^
Qualitative; interviews, case study etc.	• ↑ patient participation^44^ • ↑ reassurance^49^ • frustration^49^ • ↑ trust^52^ • ↑ relationship building^52^	• ↑ reassurance^49^	• ↑ trust^52^ • frustration^52^ • anxiety, fear^52^ • grief^52^ • loss of control^52^ • ↑ acceptance^52^		

#### Patient Reactions

Patient-centred communication, such as expressing empathy, and diagnostic reasoning strategies (e.g. explaining the diagnostic process) were associated with positive patient reactions. Patients felt reassured when doctors were empathetic and managed diagnostic expectations.^49^ Interpersonal skills, such as humour, built greater patient rapport and increased patients’ acceptance of diagnostic uncertainty.^47^

When the diagnostic process was explained to them, patients felt they could voice divergent diagnostic expectations.^51^ Patients believed doctors to be more competent and knowledgeable, and were more likely to adhere to treatment after receiving diagnostic evidence from examination^51^ or differential diagnosis instead of explicit expressions of diagnostic uncertainty.^36^ However, when doctors ruled out a serious diagnosis without providing further explanations, patients felt they needed to justify their visit.^48^

Linguistic strategies and realisations caused mixed patient reactions. While doctors believed that patients preferred diagnostic uncertainty to be omitted,^37^ patients felt frustrated if their symptoms remained unexplained and uncertainty was not addressed.^46^ Equally, for some patients, explicit disclosure of diagnostic uncertainty (e.g. ‘We don’t know what’s going on’) triggered negative emotions (e.g. fear, frustration, grief, anxiety).^52^

When doctors communicated uncertainty explicitly, patients from professional backgrounds tended to experience loss of control.^52^ Conversely, patients from lower educational backgrounds showed greater acceptance of uncertainty.^52^ When doctors openly expressed diagnostic uncertainty, listened empathetically and involved patients in planning, patients felt reassured.^49^

Additionally, patients followed explicit statements of diagnostic uncertainty with longer verbal responses indicating that these gave patients the opportunities to participate in the diagnostic interaction.^51^ Patients responded less often or not at all when doctors averted their gaze while providing a diagnosis^46^ or when they implicitly communicated their uncertainty by giving candidate diagnoses.^45^ However, if implicit uncertainty was communicated through an interrogative (e.g. ‘If I was to say to you [tentative question]’), this encouraged patients to respond and share their perspectives.^45^ No relationship was found between implicit communication of uncertainty and patient anxiety.^40^

#### Patient Experience of Care

Patient-centred communication was associated with positive patient experience of care. Explicit expressions of diagnostic uncertainty coupled with exploring patients’ emotions and listening to their concerns were associated with greater patient satisfaction.^25^ Using humour was perceived favourably and helped patients better accept diagnostic uncertainty.^47^ Understanding and managing expectations and providing plans to respond to diagnostic uncertainty were associated with trust and relationship building.^52^ Prompting patients to retell their story resulted in patients recalling facts they had not previously considered meaningful for diagnosis.^44^

Using diagnostic reasoning strategies showed mixed responses among patients. Seeking information from other clinicians, books or the internet ‘were seen as benign or even beneficial activities’^38^ to patient confidence in the doctor, while asking a nurse for help was seen as damaging to patient confidence.^38^

Overall, explicit communication of uncertainty (realised through negated statements, e.g. ‘not sure’) showed mixed results related to patients’ experience of care. Combining explicitly addressing uncertainty with patient-centred communication strategies had positive impacts on care.^25^ Epstein et al*.*^42^ did not find any association between explicit communication and a lower rating of doctor’s satisfaction, trust, autonomy, support or knowledge. Other studies reported negative patient experiences including reduced patient adherence, trust, perceived technical competence and confidence in the doctor.^36, 38^

Implicit communication and diagnostic uncertainty expressed as interrogatives (questions) or declaratives (statements) also showed mixed patient experiences. Heath^45^ found that framing the diagnosis as a question (e.g. ‘If I was to say to you…?’) promoted a positive cooperative relationship between doctors and patients by managing differences in opinion. In contrast, Ogden et al*.*^38^ found that stating diagnostic uncertainty implicitly (e.g. deferring ‘let’s see what happens’) was detrimental to patient confidence in the doctor.

No study described how omission of diagnostic uncertainty affected patient experience of care.

## DISCUSSION

This integrative review is a crucial first step in expanding our knowledge of communication strategies and linguistic expressions of diagnostic uncertainty and contributes to a small but growing evidence base of interpersonal communication in the diagnostic process.^26, 57^ To our knowledge, this is the first study to provide an evidence-based summary describing what doctors do *and say* to manage and communicate diagnostic uncertainty in primary care. We identified communicative management strategies (patient-centred and diagnostic reasoning strategies) and associated linguistic realisations (syntactic structures and lexical items) doctors commonly use when uncertain.

We found that patient-centred strategies lead to largely positive patient reactions and experience of care. Patient-centred communication strategies are known to increase patient satisfaction, improve health outcomes, enhance doctor-patient relationships and mitigate the impact of stressful situations.^58–60^ We showed that communicating uncertainty through patient-centred approaches (e.g. empathy, reassurance, humour) has similar positive effects by building better rapport between doctors and patients.

Employing diagnostic reasoning techniques, especially exclusion of serious diagnosis based on clinical test results, provided insights into the delicate nature of managing and expressing diagnostic uncertainty. Our findings show that test results can lead to apparent certainty for doctors by providing evidence to exclude a serious diagnosis yet leave patients dissatisfied as they still lack an explanation for their problem.^48^ This supports previously reported experiences of residual doubt and anxiety among patients with ongoing symptoms following a normal result.^61–64^ When doctors exclude serious diagnosis and fail to use complementary patient-centred strategies to reassure patients, patients feel required to justify their visit and ‘being sick’.^65^ Obtaining a diagnostic label can legitimise the illness and be an important part of the ‘sick role’^65, 66^ which can be denied to patients facing uncertainty. Patients, who feel doctors doubt them ‘being sick’, may become reluctant to seek medical help for the same or other health problems, with further unanticipated effects for diagnostic errors and health outcomes.^67, 68^

In practice, instead of omitting uncertainty from discussion or excluding serious diagnoses without further explanation, adopting patient-centred communication strategies alongside expressions of uncertainty could lead to greater patient satisfaction. Patient-centred approaches are particularly important in under- or misdiagnosed chronic diseases such as dementia and endometriosis as patients with uncertain or no diagnosis often feel dismissed.^69, 70^

Overall, we found mixed patient reactions and experience of care linked to linguistic expressions of diagnostic uncertainty, with implicit expressions of uncertainty better received than explicit expressions. Cultural sensitivities among patient cohorts may explain these mixed results. Meyer^52^ reported that patients from certain (unspecified) cultural backgrounds showed less tolerance for uncertainty than others. Similarly, doctors may be more or less reluctant to disclose uncertainty based on their cultural and educational background.^71, 72^

Gordon et al*.* argued that implicit linguistic expressions may be the most common form of diagnostic uncertainty but because of coding difficulty did not further investigate the distribution of these expressions in their data.^25^ Our review showed that implicit disclosure through linguistic expressions was less common than explicitly talking about uncertainty. Implicit talk occurred more often than omitting uncertainty altogether. We argue that implicit uncertainty is not just expressed through linguistic realisations and that communication strategies can also implicitly signify uncertainty.^16^ Put differently, managing uncertainty through patient-centred communication and making diagnostic reasoning more transparent for patients are also important implicit strategies to communicate uncertainty in diagnostic interactions. However, it remains unclear whether patients recognise their doctor’s uncertainty in these implicit management strategies.^16^ As diagnostic errors include failures to communicate explanations of health problems, further research drawing on authentic interactions is needed to examine if and how perception of intended message by senders (clinicians) differs from what is received and understood by patients.^16, 73–75^

This integrative review is the first to explore links between expressions of uncertainty and the ‘Big Three’ conditions (major vascular events, infections and cancers) associated with serious harms from diagnostic errors.^17^ In our review, the most frequent signs and symptoms related to two of the ‘Big Three’: infections (fever), major vascular events (chest pain) associated with acute myocardial infarction^76–78^ and neurological symptoms (dizziness) relating to stroke.^79, 80^ Our findings suggest that issues related to communication of diagnostic uncertainty frequently co-occur with presenting problems related to two ‘Big Three’ conditions. Thus, the links between expressions of uncertainty and the ‘Big Three’ require further exploration to understand how interpersonal communication might contribute to serious harm following diagnostic error.

Given the variability in communication and linguistic strategies and associated impact on patient experience of care identified in this review, we echo the multiple calls for further systematic research into how uncertainty is best communicated to patients from diverse backgrounds.^16, 26, 36, 38, 42, 52^ Health inequities due to patient characteristics, such as gender, race and language background, can be amplified by miscommunication. Our review provides a catalogue of common interpersonal communication mechanisms and expressions which further research could test and extend by investigating diagnostic interactions across different clinical contexts and patient populations.

### Limitations

Our review has several limitations. Our search was limited to three commonly used academic databases spread across medicine, psychology and linguistics and to English language articles. We did not include grey literature and may have inadvertently missed non-English scientific articles. Given the small number of included studies, we did not exclude any based on poor quality. We also had a small number of studies to draw inferences about the links between communication of diagnostic uncertainty and patient experience of care which may lower the validity of the findings. However, our extensive search strategies combined with interdisciplinary databases ensured we captured the diverse mechanisms to communicate diagnostic uncertainty.

## Conclusion

Communication of diagnostic uncertainty is pivotal in clinical practice. While communication of diagnostic uncertainty has been on the diagnostic excellence research radar, systematic investigations of actual expressions used to communicate that uncertainty have been lacking and need to be expanded. This integrative systematic review provides the first evidence-based catalogue of how diagnostic uncertainty can be expressed in primary care interactions. Results showed that doctors adopt diverse strategies and expressions to communicate uncertainty explicitly, implicitly or omit it. Our findings suggest that patients are more satisfied when patient-centred approaches are combined with diagnostic reasoning strategies to communicate uncertainty. This new knowledge can assist clinicians in primary care and beyond to increase awareness of how diagnostic uncertainty can be expressed and reflect on and potentially modify their communicative practices when facing uncertainty. This foundational information can inform further investigations to develop a more complete understanding of the relationship between expressions of diagnostic uncertainty and diagnostic errors, to reduce harm from delayed, missed or incorrect diagnosis.

## Supplementary Information

ESM 1 (DOCX 35 kb)

## Data Availability

The datasets analysed during the current study are available from the corresponding author on reasonable request.
