# Supplementary material for: Communication of Diagnostic Uncertainty in Primary Care and Its Impact on Patient Experience: an Integrative Systematic Review
Source: J Gen Intern Med. 2022 Sep 20;38(3):738–54. doi: 10.1007/s11606-022-07768-y (PMC9971421; doi:10.1007/s11606-022-07768-y)
Supplement: Supplementary file 1 — (DOCX 35 kb) [file 11606_2022_7768_MOESM1_ESM.docx]

# Appendix 1 – Search Strategies

## Medline search strategy

1. exp Communication/ or exp Health Communication/ or exp Nonverbal Communication/ or exp Professional-Patient Relations/ or exp Truth Disclosure/ or exp Communication Barriers/

2. communicat*.ti,ab.

3. 1 OR 2

4. exp Diagnosis/ or exp Delayed Diagnosis/ or exp Diagnosis, Differential/ or exp Missed Diagnosis/or exp Clinical Decision-Making/ or exp Early Diagnosis/ or exp Diagnosis, Computer-Assisted/ or exp Diagnostic Errors/

5. exp Uncertainty/

6. ("diagnostic uncertainty" or uncertainty).ti,ab.

7. ((uncertain* or imprecis* or hedge* or mitigat* or ambig* or likely or unclear or vague or question* or ruleout or explict or implicit or direct or indirect) adj4 diagnos*).ti,ab.

8. (diagnos* adj4 (doubt* or difficult* or differential or confiden* or certain*)).ti,ab.

9. ((express* adj4 uncertain*) or (communicat* adj4 Uncertain*) or (communicat* adj diagnos*)).ti,ab.

10. 5 OR 6 OR 7 OR 8 OR 9

11. Exp Primary Health Care/ or general practitioners/ or exp physicians, family/ or exp physicians, primary care/ or exp Internal Medicine/ or exp Family Practice/ or exp general practice or exp Pediatrics/ or Patients/

12. ("general practi*" or "primary care" or "family doctor*" or GP or physician* or internist* or doctor* or p?ediatric* or "general internal" or "general p?ediatric*") .ti,ab.

13. 11 Or 12

14. 3 AND 4 AND 10 AND 13

15. Limit 14 to English language

## PsycINFO search strategy

1. exp Communication/ or exp Interpersonal Communication/ or exp Interpersonal Interaction/ or exp Therapeutic Processes/ or exp Nonverbal Communication/ or exp Truth / or exp Communication Barriers/

2. communicat*.ti,ab.

3. 1 OR 2

4. exp Diagnosis/ or exp Medical Diagnosis/ or exp Differential Diagnosis/ or exp Misdiagnosis/or exp Decision-Making/ or exp Dual Diagnosis

5. exp Uncertainty/

6. ("diagnostic uncertainty" or uncertainty).ti,ab.

7. ((uncertain* or imprecis* or hedge* or mitigat* or ambig* or likely or unclear or vague or question* or ruleout or explict or implicit or direct or indirect) adj4 diagnos*).ti,ab.

8. (diagnos* adj4 (doubt* or difficult* or differential or confiden* or certain*)).ti,ab.

9. ((express* adj4 uncertain*) or (communicat* adj4 Uncertain*) or (communicat* adj diagnos*)).ti,ab.

10. 5 OR 6 OR 7 OR 8 OR 9

11. Exp Primary Health Care/ or general practitioners/ or exp family physicians / or exp Internists/ or exp Family Medicine/ or exp Pediatrics/ or Patients/

12. ("general practi*" or "primary care" or "family doctor*" or GP or physician* or internist* or doctor* or p?ediatric* or "general internal" or "general p?ediatric*") .ti,ab.

13. 11 OR 12

14. 3 AND 4 AND 10 AND 13

15. Limit 14 to English language

## Linguistics and Language Behaviour Abstracts (LLBA) search strategy

1. MAINSUBJECT.EXPLODE("Truth") OR MAINSUBJECT.EXPLODE("Dyadic Interaction") OR MAINSUBJECT.EXPLODE("Practitioner Patient Relationship") OR MAINSUBJECT.EXPLODE("Interpersonal Behavior") OR MAINSUBJECT.EXPLODE("Interpersonal Relationships") OR MAINSUBJECT.EXPLODE("Nonverbal Communication") OR MAINSUBJECT.EXPLODE("Interpersonal Communication") OR MAINSUBJECT.EXPLODE("Communication") OR MAINSUBJECT.EXPLODE("Workplace Communication") OR MAINSUBJECT.EXPLODE("Negotiation") OR AB,TI(communicat*)
2. MAINSUBJECT.EXPLODE("Diagnosis") OR MAINSUBJECT("Diagnostic Tests") OR MAINSUBJECT.EXPLODE("Cognitive Processes") OR MAINSUBJECT.EXPLODE("Judgment") OR AB,TI(diagnos*) OR AB,TI("decision-making") OR AB,TI (misdiagnos*) OR AB,TI("diagnostic error") OR AB,TI("clinical reasoning")
3. (("diagnostic uncertainty") OR (uncertainty) OR (diagnos*)) AND ((imprecis*) OR (hedge*) OR (mitigat* ) OR (ambig*) OR (likely) OR (unclear ) OR (vague) OR (question* N) OR (ruleout) OR (explicit) OR (implicit) OR (direct ) OR (indirect) OR (difficult*) OR (differential) OR (confiden*) OR (express*) OR (certain) OR (uncertain)) OR MAINSUBJECT.EXPLODE("Hedges/Hedging")
4. MAINSUBJECT.EXPLODE("Health Care Practitioners") OR MAINSUBJECT.EXPLODE("Physicians") OR MAINSUBJECT.EXPLODE("Language for Special Purposes") OR MAINSUBJECT.EXPLODE("Medical Language") OR MAINSUBJECT.EXPLODE("Medicine") OR AB,TI("general practi*") OR AB,TI("primary care") OR AB,TI("family doctor*") OR AB,TI(GP) OR AB,TI(physician*) OR AB,TI(internist*) OR AB,TI(doctor*) OR AB,TI(p?ediatric*) OR AB,TI("general internal")

5. S1 AND S2 AND S3 AND S4

6. Limit S5 to English

# Appendix 2 – Inclusion and exclusion criteria

**Participants/population**

**Inclusion population:**

• Clinic-based primary care settings including general practice /family medicine, general internal medicine, general paediatric medicine.

• Medical students, primary care trainees and specialists involved in delivery or training for providing primary care

• Patients in primary care visits

• Family and/or carers involved in primary care visits

**Exclusion population:**

• Other hospital-based primary care and non-primary care setting, e.g. emergency, residential care, home care, telehealth.

• Other primary care clinicians such as nurses, pharmacists, dentists, emergency.

**Studies**

**Inclusion:**

• Studies/publications describing actual or simulated communication of diagnostic uncertainty in primary care settings

• Studies/publications describing the experiences and attitudes of doctors and patient towards

communication of diagnostic uncertainty

**Exclusion:**

• Studies not in English, government reports or policy document, editorials, opinion pieces, reviews, conference abstract, full length books, thesis and media reports.

• Studies describing clinician-clinician interactions related to communication of uncertainty

• Studies describing communication of uncertainty unrelated to diagnosis, e.g. prognosis, therapy, management, public discourse

Studies without an explicit focus on communication aspect of diagnostic uncertainty (e.g. how to clinically manage diagnostic uncertainty)

• Studies describing communication of diagnostic uncertainty by other primary health care providers such as nurse practitioners, dentist pharmacists, allied health professionals

• Studies describing written communication related to diagnostic uncertainty (e.g. in electronic health records, pathology or imaging reports, referral or specialist letters)

• Studies describing of diagnostic communication without mention of uncertainty

# Appendix 3 – QATSDD Scores

| Author | year | TOTAL QATSSD Score in % | Explicit theoretical framework (0-3) | Statement of aims /objectives in main body of report (0-3) | Clear description of research setting (0-3) | Evidence of sample size considered in terms of analysis (0-3) | Representative sample of target group of a reasonable size (0-3) | Description of procedure for data collection (0-3) | Rationale for choice of data collection tool(s) (0-3) | Detailed recruitment data (0-3) | Statistical assessment of reliability and validity of measurement tool(s) (Quantitative only) (0-3) | Fit between stated research question and method of data collection (Quantitative) (0-3) | Fit between stated research question and format and content of data collection tool e.g. interview schedule (Qualitative only) (0-3) | Fit between research question and method of analysis (0-3) | Good justification for analytical method selected (0-3) | Assessment of reliability of analytical process (Qualitative only) (0-3) | Evidence of user involvement in design (0-3) | Strengths and limitations critically discussed (0-3) |
| --- | --- | --- | --- | --- | --- | --- | --- | --- | --- | --- | --- | --- | --- | --- | --- | --- | --- | --- |
| Arborelius | 1991 | 33.3 | 0 | 3 | 2 | 0 | 3 | 1 | 0 | 0 |  |  | 2 | 2 | 0 | 1 | 0 | 0 |
| Bhise | 2018 | 73.8 | 3 | 3 | 2 | 3 | 0 | 3 | 3 | 1 | 1 | 2 |  | 2 | 2 |  | 3 | 3 |
| Clarke | 2014 | 83.3 | 2 | 3 | 3 | 3 | 2 | 3 | 3 | 3 |  |  | 3 | 2 | 2 | 3 | 0 | 3 |
| Cousin | 2013 | 77.1 | 3 | 3 | 3 | 0 | 2 | 3 | 3 | 2 | 1 | 2 | 3 | 2 | 3 | 2 | 2 | 3 |
| Epstein | 2007 | 85.4 | 3 | 3 | 3 | 1 | 2 | 3 | 3 | 3 | 2 | 2 | 3 | 3 | 2 | 3 | 2 | 3 |
| Gerrity | 1990 | 88.1 | 3 | 3 | 3 | 0 | 3 | 2 | 3 | 3 | 3 | 3 |  | 3 | 3 |  | 2 | 3 |
| Gerrity | 1992 | 76.2 | 3 | 3 | 3 | 0 | 3 | 2 | 3 | 3 | 1 | 2 |  | 3 | 2 |  | 2 | 2 |
| Gordon | 2000 | 58.3 | 2 | 3 | 2 | 0 | 2 | 2 | 2 | 1 | 0 | 2 | 2 | 2 | 2 | 1 | 2 | 3 |
| Heath | 1992 | 19.0 | 3 | 2 | 2 | 0 | 0 | 1 | 0 | 0 |  |  | 0 | 0 | 0 | 0 | 0 | 0 |
| Heritage | 2019 | 42.9 | 3 | 3 | 3 | 0 | 0 | 1 | 0 | 0 |  |  | 2 | 2 | 2 | 1 | 0 | 1 |
| Maynard | 2006 | 26.2 | 3 | 2 | 2 | 0 | 0 | 0 | 0 | 0 |  |  | 2 | 2 | 0 | 0 | 0 | 0 |
| Maynard | 2003 | 23.8 | 2 | 2 | 0 | 1 | 0 | 0 | 2 | 0 |  |  | 2 | 1 | 0 | 0 | 0 | 0 |
| Meyer | 2019 | 78.6 | 3 | 3 | 3 | 0 | 3 | 3 | 1 | 3 |  |  | 3 | 2 | 2 | 3 | 1 | 3 |
| Ogden | 2002 | 76.2 | 3 | 3 | 3 | 0 | 2 | 1 | 2 | 3 | 2 | 2 |  | 3 | 3 |  | 2 | 3 |
| Olsen | 2018 | 83.3 | 3 | 3 | 3 | 0 | 3 | 3 | 2 | 3 | 3 | 3 |  | 3 | 3 |  | 1 | 2 |
| Paton | 2017 | 23.8 | 1 | 2 | 1 | 1 | 1 | 0 | 0 | 0 |  |  | 0 | 2 | 2 | 0 | 0 | 0 |
| Peräkylä | 1998 | 42.9 | 3 | 2 | 2 | 0 | 1 | 1 | 2 | 0 |  |  | 2 | 2 | 2 | 0 | 0 | 1 |
| Peräkylä | 2006 | 38.1 | 3 | 2 | 2 | 0 | 1 | 1 | 0 | 0 |  |  | 2 | 2 | 2 | 0 | 0 | 1 |
| Stortenbeker | 2019 | 57.1 | 2 | 3 | 1 | 1 | 2 | 2 | 1 | 1 | 2 | 2 |  | 2 | 2 |  | 0 | 3 |
| Wiener | 2013 | 64.3 | 3 | 2 | 2 | 2 | 1 | 2 | 2 | 2 |  |  | 2 | 2 | 2 | 3 | 0 | 2 |
